# Through a glass darkly: facial wrinkles affect our processing of emotion in the elderly

**DOI:** 10.3389/fpsyg.2015.01476

**Published:** 2015-10-01

**Authors:** Maxi Freudenberg, Reginald B. Adams, Robert E. Kleck, Ursula Hess

**Affiliations:** ^1^Department of Psychology, Social and Organizational Psychology, Humboldt-Universität zu BerlinBerlin, Germany; ^2^Department of Psychology, The Pennsylvania State UniversityUniversity Park, PA, USA; ^3^Department of Psychological and Brain Sciences, Dartmouth CollegeHanover, NH, USA

**Keywords:** face perception, emotional expression, face age, signal clarity, visual search task

## Abstract

The correct interpretation of emotional expressions is crucial for social life. However, emotions in old relative to young faces are recognized less well. One reason for this may be decreased signal clarity of older faces due to morphological changes, such as wrinkles and folds, obscuring facial displays of emotions. Across three experiments, the present research investigates how misattributions of emotions to elderly faces impair emotion discrimination. In a preliminary task, neutral expressions were perceived as more expressive in old than in young faces by human raters (Experiment 1A) and an automatic system for emotion recognition (Experiment 1B). Consequently, task difficulty was higher for old faces relative to young faces in a visual search task (Experiment 2). Specifically, participants detected old faces expressing negative emotions less accurately and slower among neutral faces of their peers than young faces among neutral faces of their peers. Thus, we argue that age-related changes in facial features are the most plausible explanation for the differences in emotion perception between young and old faces. These findings are of relevance for the social interchange with the elderly, especially when multiple older individuals are present.

## Introduction

Would you rather approach someone who looks happy or mad? Because individuals live in and depend on groups, we take note of each other’s emotional expressions and react in accordance with the information provided by them. Hence, we are likely to approach someone who shows happiness, as this emotion signals that the person is pleased with the current situation. In contrast, if we perceive anger, we probably do not wish to spend time with the angry other, and therefore avoid interaction. Thus, emotional expressions can serve as an important means of communication (Parkinson, 1996) and convey distinct social signals that can have important effects on the social behavior of others (Van Kleef et al., 2010; Hareli and Hess, 2012; Weisbuch and Adams, 2012). Yet, for the perceiver to benefit from the information conveyed by the emotional displays of others and to react with the most adaptive social behavior, these displays first have to be decoded successfully.

Overall, people are rather good at decoding facial expressions of emotion, especially with the highly prototypical ones used in most research to date (Tracy and Robins, 2008; Hess and Thibault, 2009). Nevertheless, the relationship between the encoder, the person who displays an emotion, and the decoder, the one interpreting the facial expression, can impact decoding accuracy. Thus, emotions expressed by members of the same culture (see Elfenbein and Ambady, 2002, for a meta-analysis) or social in-group members (Thibault et al., 2006) are better recognized. Even bogus group membership operationalized as apparent personality type affects decoding accuracy (Young and Hugenberg, 2010).

The relative ages of the encoder and decoder also play a role in emotion recognition. The literature suggests reduced decoding accuracy for old faces relative to young faces by young decoders, but also by older participants (see Fölster et al., 2014, for a review). One explanation for this is that old encoders are simply poorer communicators, as their aged facial muscles constrain the ability to express emotions intensely (Malatesta et al., 1987b). Even though older participants generally self-report less expressive behavior for negative emotions, empirical evidence regarding emotional expressivity as a function of age is mixed (Gross et al., 1997). Age differences in emotional expressivity may have their origin in differences in emotional experience. Thus, when asked about the emotions they show in general, older participants report less negative emotions. For example, older nuns report less negative and more positive emotions relative to their younger peers (Gross et al., 1997). By contrast, when young, middle-aged and old women in another study were asked to recall a specific emotional experience, no age-related differences in the self-rated intensity level of their emotional arousal were found (Malatesta et al., 1987b). In addition, age is associated with increased emotional control (Gross et al., 1997). The authors distinguish between inner control, which targets emotional experience and external control, which regulates expressive behavior. They further argue that individuals learn over a lifetime of experience to effectively regulate primarily the inner experience of emotion.

If the lower decoding accuracy for older faces were an artifact of age-related changes in facial musculature or emotional reactivity, it should disappear when expressions are equated for intensity. However, the effect maintains when such artificial stimuli are used (Hess et al., 2012). The authors therefore propose that the difference in decoding accuracy is linked to the decreased signal clarity of older faces. Specifically, the complexity of facial features increases in the aging face due to age-related changes such as wrinkles and folds. Once developed, those features, independent of the situation or emotional context, can emphasize some emotional expressions but also obscure others. Hence, structural changes in the old face may blur the clarity of an emotional signal (Malatesta et al., 1987b). As a consequence, emotional displays in older faces are more ambiguous than in young faces, making it more difficult to identify the emotional state underlying the facial expression of older adults (e.g., Ebner and Johnson, 2009).

The present article aims to contribute to the understanding of the mechanisms that underpin differences in emotion perception as a function of face age in three experiments. Our general assumption is that the misattribution of emotions to elderly faces (Experiments 1A,B) creates impairment in emotion discrimination, particularly when multiple individuals are present (Experiment 2). To investigate emotion discrimination in young and old faces, researchers typically present facial expressions individually and ask participants to either judge the face using forced-choice scales or rate the emotion expression on multiple intensity scales. In Experiment 1A, we make use of the latter approach to complement findings on the misattribution of emotions in the elderly face. However, instead of presenting emotionally expressive faces, we investigate how emotion perception in solely neutral faces varies as a function of expresser age. In Experiment 1B, we collect emotion judgments on the same stimuli by a software tool for automatic facial expression recognition. For both procedures, we hypothesize that neutral faces of the elderly are perceived as less neutral than neutral young faces, because the wrinkles and folds of older faces will appear “emotional.” If indeed the neutral faces of the elderly appear to convey emotions, then it should be the case that it is more difficult to distinguish an emotional old face from emotionally neutral old faces than to do the same task for young faces. A novel way to test this is to use a visual search task in which an emotional old or young face is embedded into an array of neutral faces of their peers. We do this in Experiment 2. An additional advantage of this paradigm is that participants are presented with a group of individuals. Thus, we can examine emotion discrimination in a way that is closer to real life situations, which provides more ecological validity.

In sum, as the correct identification of facial emotional displays is essential for a successful social life, the goal of the present article is to assess whether the impairment of emotion perception in elderly faces is due to a lack in signal clarity. The focus of Experiment 1, as a preliminary study, is to extend previous findings that aging degrades the signal value of emotional expressions, by examining whether the elderly faces are perceived as less neutral than young faces even in their neutral displays. This notion is explored more fully in that we complement findings on human ratings (Experiment 1A) with the results of an automated system for facial expression recognition (Experiment 1B). The focus then of Experiment 2, is to examine the effect of face age on emotion perception in a visual search task. If neutral old faces are perceived as more emotionally expressive, we predict that neutral expressions in older faces will be more distracting when the task is to find an emotional face within an array of neutral faces. Thus, we expect that older faces depicting happiness, anger or sadness will be identified more slowly and less accurately within old-age-groups relative to young faces in young-age-groups.

## Experiment 1A

Experiment 1A was designed to investigate how the age of a face influences the perception of emotions in neutral faces and show that neutral facial displays of the elderly are perceived as less neutral than neutral young faces. In this regard, we defined a neutral face as a facial expression that displays a neutral state, when no emotional expression is intended by the expresser. Despite this objective neutrality, participants are known to misattribute emotions to the elderly face, when a forced-choice format is applied (Malatesta et al., 1987a). For methodical reasons of assessing neutrality via a rating scale, we take an indirect route by asking participants to rate the expression intensity of the facial display on multiple emotion scales. If a face is perceived as indeed neutral, ratings on the emotion scales should be low. Specifically, we expected participants to attribute more emotionality to the elderly relative to young individuals.

### Materials and Methods

#### Participants

The study was carried out in accordance with the procedures approved by the Humboldt-Universität zu Berlin Psychology Department ethics committee. Potential participants were invited to take part in an online survey through a newsletter from the Humboldt-Universität zu Berlin. In the introduction to the online survey, interested volunteers were informed concerning the objectives and procedures of the investigation. Preservation of their anonymity was guaranteed through the data collection methods that were employed. Individuals were told that completing the survey would constitute their informed consent to participate in the study. Upon completion of the online survey, participants had the option to demand the deletion of their data and thus to withdraw from the study. No individual made use of this option. A total of 45 (10 men) participants, primarily students, completed the survey. They had a mean age of 24.84 years (*SD* = 5.17), ranging from 18 to 47 years.

#### Materials

Stimuli were chosen from a larger database of color photographs of faces (Ebner et al., 2010) of female and male actors varying in age. The subset of images selected for the present experiment included only neutral expressions by 36 either young (19–31 years) or old (69–80 years) men and women. Both sexes were equally represented. The order of presentation was randomized.

#### Procedure and Dependent Variables

While seeing the neutral facial picture on a computer screen for as long as they wanted, participants rated the emotion expression on each of the following 7-point scales anchored between 0 (“not at all”) and 6 (“very intense”): Anger, sadness, happiness, fear, disgust, surprise, and contempt. Although all images depicted individuals with neutral facial expressions, we expected the participants to perceive the expressions as emotional. Hence, participants were encouraged to inspect the images carefully as the face rating task would be harder for some faces than for others.

### Results and Discussion

To determine whether participants perceive older compared to younger neutral faces as more expressive as a function of face age, emotion rating and sex, mean intensity ratings for each scale were computed for each expresser type. A three-way analysis of variance (ANOVA), with face age (young vs. old), emotion (anger, sadness, happiness, fear, disgust, surprise, and contempt), and face sex (female vs. male) all as within-subjects factors was conducted. Where Mauchly’s test indicated the violation of sphericity, Greenhouse Geisser corrections were applied and degrees of freedom were rounded to the nearest integer.

**FIGURE 1:**
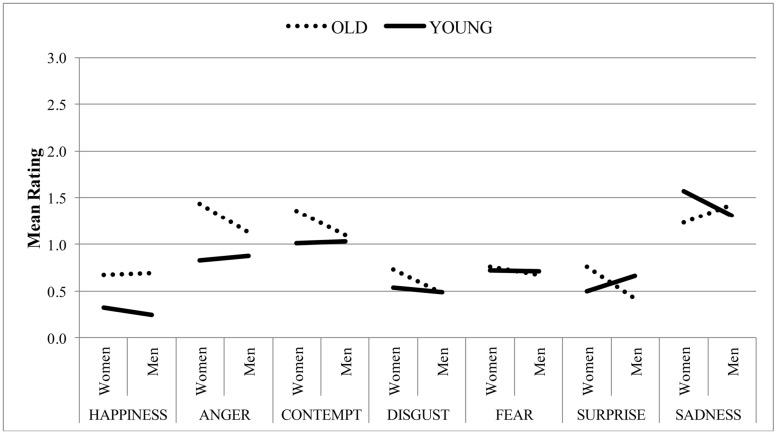
**Mean intensity ratings on the emotion scales as a function of face age and sex by human raters**.

As predicted, the analysis revealed a main effect of face age, *F*(1,44) = 25.56, *p <* 0.001, $\eta_{p}^{2}$ = 0.37, such that overall, neutral old faces (*M* = 0.92, *SE* = 0.10) were rated as more expressive than neutral young faces (*M* = 0.77, *SE* = 0.10). However, this effect was qualified by a significant face age × sex × emotion interaction (see **Figure 1**), *F*(4,183) = 13.18, *p* < 0.001, $\eta_{p}^{2}$ = 0.23. The main effects of sex and emotion as well as the face age × sex and face age × emotion interactions reached significance, but were also qualified by the three-way interaction [sex: *F*(1,44) = 12.74, *p <* 0.001, $\eta_{p}^{2}$ = 0.23; emotion: *F*(3,117) = 30.03, *p <* 0.001, $\eta_{p}^{2}$ = 0.41, face age × sex: *F*(1,44) = 9.64, *p* = 0.003, $\eta_{p}^{2}$ = 0.18, face age × emotion: *F*(4,155) = 12.96, *p <* 0.001, $\eta_{p}^{2}$ = 0.23].

To decompose the three-way interaction, we conducted simple effects analyses in the form of separate ANOVAs on the individual emotion scales. For ratings of *happiness*, a main effect of face age emerged, *F*(1,44) = 90.36, *p* < 0.001, $\eta_{p}^{2}$ = 0.67, such that participants rated neutral old faces (*M* = 0.68, *SE* = 0.07) as more intensely happy than neutral young faces (*M* = 0.28, *SE* = 0.06). For ratings of *anger* as well, a main effect of face age emerged, *F*(1,44) = 31.29, *p* < 0.001, $\eta_{p}^{2}$ = 0.42, such that participants also attributed more anger to neutral old faces (*M* = 1.28, *SE* = 0.14) relative to neutral young faces (*M* = 0.85, *SE* = 0.14). However, there was also a significant main effect of sex, *F*(1,44) = 4.64, *p* = 0.04, $\eta_{p}^{2}$ = 0.10, which was qualified by a significant face age × sex interaction, *F*(1,44) = 17.26, *p <* 0.001, $\eta_{p}^{2}$ = 0.28. Whereas neutral old women were perceived as angrier than old men, *t*(44) = 3.45, *p* = 0.001, there was no difference between neutral young women and young men. For ratings of *contempt*, there was also a significant main effect of face age, *F*(1,44) = 6.981, *p* = 0.01, $\eta_{p}^{2}$ = 0.14, as well as a marginally significant main effect of sex, *F*(1,44) = 3.59, *p* = 0.06, $\eta_{p}^{2}$ = 0.07. Both effects were qualified by a significant face age × sex interaction, *F*(1,44) = 4.97, *p* = 0.03, $\eta_{p}^{2}$ = 0.10, such that again old women were perceived as more contemptuous relative to young women, *t*(44) = 2.99, *p* = 0.01, but also relative to old men, *t*(44) = 2.52, *p* = 0.02. Similarly, for ratings of *disgust*, there was a marginally significant main effect of face age, *F*(1,44) = 3.18, *p* = 0.08, $\eta_{p}^{2}$ = 0.07, and a significant main effect of sex, *F*(1,44) = 17.77, *p <* 0.001, $\eta_{p}^{2}$ = 0.29, that were both qualified by a significant face age × sex interaction, *F*(1,44) = 5.76, *p* = 0.02, $\eta_{p}^{2}$ = 0.12. Again, neutral old women were perceived as more disgusted than young women, *t*(44) = 2.57, *p* = 0.01, or old men, *t*(44) = 4.86, *p < 0*.001. For ratings of *surprise*, a significant main effect of sex emerged, *F*(1,44) = 6.83, *p* = 0.01, $\eta_{p}^{2}$ = 0.13, that was qualified by a significant face age × sex interaction, *F*(1,44) = 27.47, *p* < 0.001, $\eta_{p}^{2}$ = 0.38. In line with the other emotion ratings, participants attributed more surprise to neutral old women relative to young women, *t*(44) = 3.57, *p* = 0.001, or old men, *t*(44) = 5.86, *p* < 0.001. Contrariwise, old men were rated as less surprised relative to young men, *t*(44) = -3.83, *p* < 0.001. Furthermore, compared to young men, young women were perceived as less surprised, *t*(44) = -2.66, *p* = 0.01. For ratings of *fear*, neither the main effects of face age or sex nor the face age × sex interaction reached significance. As a last point, for ratings of *sadness*, there was a marginally significant effect of face age, *F*(1,44) = 3.51, *p* = 0.07, $\eta_{p}^{2}$ = 0.07, qualified by a significant face age × sex interaction, *F*(1,44) = 25.53, *p* < 0.001, $\eta_{p}^{2}$ = 0.37. In contrast to all the other emotion ratings, old women were perceived as less sad relative to young women, *t*(44) = -4.48, *p* < 0.001, and old men, *t*(44) = -2.58, *p* = 0.01. Then again, more sadness was marginally attributed to neutral faces of old men relative to young men, *t*(44) = 1.68, *p* = 0.099. Furthermore, more sadness was attributed to neutral faces of young women compared to young men, *t*(44) = 3.52, *p* = 0.001. All other effects were not significant.

In sum, our results replicate other findings that neutral old faces are perceived as more emotionally expressive than are young faces (Hess et al., 2012), which is consistent with the conclusion that some of the wrinkles and folds that develop as a function of aging are perceived as emotional cues in the face. Given that participants judged neutral expressions, it is not at all surprising that the ratings on the individual emotions were generally low. In other research, when a forced-choice format was used in order to select a discrete emotional expression, accuracy rates for neutral facial displays were lower for old relative to young faces (Ebner and Johnson, 2009). This is in line with our overall finding that neutral old faces were perceived as more expressive than young faces. Further, for old faces Malatesta et al. (1987a) showed that most errors relate to the misattribution of sadness, contempt and anger. The same pattern occurred in our sample, but also extended to misattributions of these emotions in the young face. With regard to the individual emotion ratings, we replicated the finding that neutral faces of the old are perceived as angrier than neutral young faces (Hess et al., 2012), but also found higher intensity ratings for happiness for the elderly. Further, in the current sample, there was no difference between young and old neutral faces for fear. However, relative to young faces, more contempt, disgust, and surprise was misattributed to neutral faces of old women and also more sadness to faces of old men.

Though we are here advancing the notion that age-related changes in the face are responsible for how age of the face may affect emotional decoding alternative explanations need to be acknowledged (see Fölster et al., 2014, for a review). These include age-related differences in the production of facial expressions. However, as all faces depicted a neutral expression, less controllability of muscle tissues with age seems an unlikely explanation for the finding that human raters judge neutral faces of the elderly as more expressive than neutral faces of young individuals.

In Experiment 1A, people rated neutral faces of young and old individuals on emotion intensity. This procedure allowed us to investigate whether the elderly overall might be perceived as less neutral. However, one limitation of this approach is that age-related differences in a neutral expression are derived only indirectly through intensity ratings on discrete emotion scales. Hence, next it seems desirable to assess neutrality directly in form of a discrete value, which will be realized in Experiment 1B. Further, Experiment 1A does not address whether the misattributions to neutral faces of the elderly indeed result from age-related changes in the face or, alternatively, relate to stereotype knowledge. Such stereotypes have previously been reported to bias emotion perception (see Fölster et al., 2014, for a review). Thus, in Experiment 1B, we used an approach that is independent of stereotype knowledge by submitting the faces to an automated facial expression recognition system.

## Experiment 1B

### Materials and Methods

The same neutral stimuli as were used in Experiment 1A were submitted to the computer expression recognition toolbox (CERT; Littlewort et al., 2011). CERT is a software tool for automatic facial expression recognition. The program registers the intensity of different facial action units in individual images. Given these as inputs, CERT also creates probability estimates for prototypical facial emotion expressions. Correlations between the human ratings and probability estimates by CERT can be downloaded as Supplementary Material.

### Results and Discussion

**FIGURE 2:**
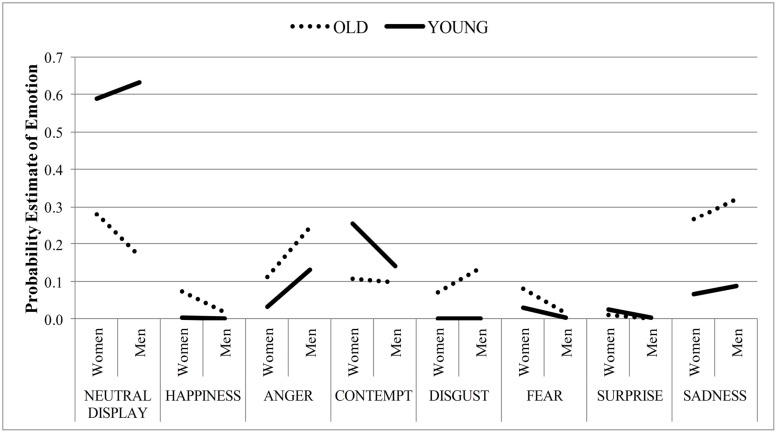
**Mean probability estimate of emotion expression as a function of face age and sex by the automated emotion recognition system**.

**Figure 2** shows the probability estimates for each emotion and a neutral facial expression based on the registered facial action units in young and old faces of men and women by CERT. We conducted two-way independent ANOVAs on the probability estimates for each emotion and the neutral facial expression with face age and face sex as between factors.

As predicted, the analysis on the probability estimates for *neutrality* revealed a main effect of face age, *F*(1,32) = 45, *p* < 0.001, $\eta_{p}^{2}$ = 0.58, such that old faces were coded as less neutral than young faces. As such, even the automated emotion recognition system attributes less neutrality to old faces relative to young faces, when the face actually depicts a neutral expression. It should be noted that the probability estimates for each emotion are mutually dependent such that a low probability estimate of a correct neutral facial expression consequently is linked to higher probability estimates in all remaining erroneous cases. Thus, in case of an old face relative to a young face, the computational analysis estimates the probability that the neutral face expresses *disgust*, *F*(1,32) = 9.22, *p* = 0.01, $\eta_{p}^{2}$ = 0.22, *sadness*, *F*(1,32) = 11.16, *p* = 0.002, $\eta_{p}^{2}$ = 0.26, and *happiness* as more likely, *F*(1,32) = 7.42, *p* = 0.01, $\eta_{p}^{2}$ = 0.19. However, the computational analysis also reveals that neutral young faces express *contempt* more than neutral old faces, *F*(1,32) = 5.27, *p* = 0.03, $\eta_{p}^{2}$ = 0.14.

Further, the main effect of face sex reached significance for the probability estimates in neutral faces of *fear*, *F*(1,32) = 4.85, *p* = 0.04, $\eta_{p}^{2}$ = 0.13, *surprise*, *F*(1,32) = 4.95, *p* = 0.03, $\eta_{p}^{2}$ = 0.58, as well as marginally for *happiness*, *F*(1,32) = 3.45, *p* = 0.07, $\eta_{p}^{2}$ = 0.10, and *anger*, *F*(1,32) = 3.93, *p* = 0.06, $\eta_{p}^{2}$ = 0.11. Specifically, it seems more likely that the neutral face of a women signals more fear, surprise and happiness, but also less anger than a man’s neutral face. All other effects, inclusive of all age × sex interactions, were not significant.

The findings from Experiment 1A indicated a higher overall expressiveness in the neutral old face relative to the neutral young face. Experiment 1B offered a complementary finding, such that even an automated emotion recognition system ascribes neutral old faces less neutrality than neutral young faces. Alternatively, perceiving emotions in neutral faces may be considered as misjudgments. Comparing the specific types of errors toward neutral faces, similarities between the two empirical procedures emerge, such as the finding that human raters as well as CERT misattributed more happiness to the neutral faces of the elderly relative to young faces. Moreover, most age-related differences in the probability estimates by the automated recognition tool in Experiment 1B are in line with the human ratings in Experiment 1A, at least in subgroups by face sex, e.g., the higher disgust rating in old relative to young women’s faces. However, some differences in the misattribution of emotions to the neutral face occurred as well between the two experimental designs. This suggests that the wrinkles and folds render the face more ambiguous. Given such an ambiguous face, it remains possible that stereotypical knowledge about the elderly may be used as a cue. However, age information alone does not lead to an activation of stereotypic traits (Casper et al., 2011). More importantly, age-related beliefs about emotion expression are highly heterogeneous (Montepare and Dobish, 2013). As such, divergent findings between the two experiments may also be linked to two other possibilities. First, the procedural variations and accompanying scope varied between the two experiments. In Experiment 1A participants judged intensity levels of emotions. Thereby, the different ratings on the emotion scales are rather independent from one another. In contrast, the automated assessment is similar to a recognition task. Hence, the probability estimates of emotions computed by CERT depend on each other in an additive relation. Secondly, both experiments indicated that face sex also influences emotion recognition, either by itself, or in interaction with face age. Generally, facial appearance, especially structural differences between male and female faces have been known to influence emotion perception, e.g., anger and happiness (Hess et al., 2004; Becker et al., 2007; Adams et al., 2015). In the course of aging, women are then more affected by age-related changes (see Albert et al., 2007, for a review). Specifically, women tend to develop more and deeper wrinkles in the perioral region than men due to differences in skin appendages, precisely fewer sebaceous glands, sweat glands, and blood vessels (Paes et al., 2009). Apart from wrinkles as the most prominent age-related change, loss of tissue elasticity and facial volume also follow age-related changes in bony support structure (see Albert et al., 2007, for a review). This might lead to an overall more concave look that might contribute to lower signal clarity, especially for old women. Then again, the CERT as an automated tool for emotion detection in faces was developed to register the activation pattern of facial muscles that are associated with emotion expression. However, individual raters are not confined to this and process a wider range of facial signals, even perhaps the hairstyle of an individual. In this vein, facial cues of dominance and affiliation are also known to affect emotion attribution in human raters (Hess et al., 2010). Thus, the fact that the computational analysis is restricted to differential activation of facial muscles, whereas human raters process facial characteristics in its entirety, might possibly explain the face age by sex interactions for human raters, which was not significant for the automated assessment.

We expected to find that participants would perceive neutral old faces as less neutral than neutral young faces. This is supported by the finding of an overall main effect of face age (Experiments 1A,B) such that faces of the elderly would be perceived as overall more emotional than those of young individuals. When it comes to the level of specific emotions, it can be argued, that not all discrete emotions reveal an effect of face age, apparently questioning our proposition of impaired signal clarity due to age-related changes in a face. However, one important thing to note is that we did not hypothesize emotion specific biases in the misattribution of emotions to neutral old faces for several reasons. First, the overlap between age-related changes in a face and expressive facial markers varies highly between distinct emotions. As such, emotions are more likely affected by misattributions, if the expressive markers of that emotion actually correspond to facial wrinkles and folds that develop in the course of aging, as for example the facial display of anger and frown lines on the forehead. In contrast, emotions like surprise and fear that are characterized by the opening of the mouth and eyes (Tomkins and McCarter, 1964) are less likely to be misperceived in young and old faces. Accordingly, neither the human rating nor the computational analysis revealed an age of face effect for fear to a neutral face. On a different note, facial wrinkles and folds are an especially idiosyncratic feature. Normally, in one’s early 20 s fine facial lines start to emerge, for example horizontally across the forehead or vertically between the eyebrows (Albert et al., 2007). Given a certain age level, individuals naturally share the characteristic of manifested facial wrinkles in general. Yet, differences in the specific type of lines are apparent between individuals, as for example the nasolabial groove, glabellar lines, or crow’s feet. In fact, there is even evidence for some linkage between the emotion traits of the elderly and emotion resembling aging wrinkles and folds (Malatesta et al., 1987a).

In sum, despite some differences between the human ratings and the computational analysis in the specific misattributions to old and young faces that were found, both procedures found that neutral faces of the elderly are perceived as less neutral and thus more emotionally expressive than faces of young individuals. This renders stereotypical expectations as the sole underlying mechanism unlikely and supports our principal hypothesis that age-related changes in a face impair the signal clarity of emotion expression.

## Experiment 2

In Experiments 1A,B, we found that neutral old faces resemble emotional faces more than do young faces, probably as a function of age-related changes in facial appearance. This difference should impair performance in a visual search task, where participants have to find an emotional face among multiple neutral faces presented concurrently. Specifically, if the neutral faces of the elderly are perceived as more expressive, as was the case in Experiment 1, this should serve to increase the level of difficulty in a visual search task. As a variation on the standard paradigm employed in this sort of research, participants not only had to decide whether one expression is different from the others or whether all are the same, but were asked to locate the position of the one emotional stimulus among neutral faces of the same age and sex group. This strategy removes the need for non-target trials and thus reduces the number of trials a participant must complete. It also allows us to know which specific expression was perceived to be emotional. As all neutral expressions can be seen as expressing some, low level, of emotion, this reduces the risk of false positives. We then compared response times and accuracy rates to determine, whether one stimulus type is recognized faster than another as a function of the target emotion. The literature on visual search paradigms of this sort so far shows that discrepant targets are found more quickly among neutral distractors than among emotional ones (Pinkham et al., 2010). Further, search efficiency in visual search tasks is improved the more similar the distractor items are and the more the target item deviates from distractor items (Duncan and Humphreys, 1989). Hence, as neutral old distractor faces appear less neutral and resemble emotional states more (Experiments 1A,B), we predict better overall expression identification for young faces. Thus, we predict for Experiment 2 that finding a young emotional face among multiple young neutral faces will be faster and more accurate than finding an old emotional face among multiple old neutral faces.

### Materials and Methods

#### Participants

A total of 51 volunteers from the Berlin area were tested in groups of up to six. The study was approved by the Humboldt-Universität zu Berlin Psychology Department ethics committee. After verbal consent was given, participants worked on individually assigned computer workstations, separated by partitions. Subsequent to the experiment, participants were given feedback on their individual performance plus a short presentation on the current state of research as well as the object of this investigation. Participants were then asked for their written permission to use their data. Four participants refused permission and their data were deleted. Hence, the final sample consisted of 19 men and 28 women ranging in age from 14 to 65 years with a mean age of 35 (*SD* = 13.93) years. Data from an additional four participants were discarded from the analysis due to an error rate higher than 25% of trials in at least one of the three emotion blocks.

#### Materials

As in Experiment 1, images were chosen from the FACES database (Ebner et al., 2010). The stimulus set for the visual search task consisted of 144 photos from 36 identities (nine per age and sex group), each displaying a happy, angry, sad, and neutral facial expression. Participants saw an equal number of young (19–31 years) and older (69–80 years) male and female faces.

#### Design and Dependent Variables

Pictures were presented and responses recorded via E-Prime 2.0 software. To create a realistic “group,” 9 images of multiple identities from the same sex and age group were presented simultaneously on a 3 by 3 matrix for each trial. Within the 3 by 3 matrix, the target face was equally often presented at each position. All distractor faces showed neutral expressions. Overall, there were three blocks of trials that differed in the target emotions (happiness, sadness, or anger). Block presentation was counterbalanced. Within each emotion block, trials varied randomly in terms of target sex and age, such that each target group appeared nine times per emotion block. This resulted in a 3 (target emotion) × 2 (target age) × 2 (target sex) × 9 (target position) design. At the beginning of each emotional block, participants were informed about the type of target emotion, e.g., “Where is the happy face?” and completed four practice trials with different actors than those used in the main experiment. These practice trials had the purpose of introducing the task design and target emotion to the subjects. Following each practice trial, the respective emotion block was completed. Responses were made by moving and clicking the computer mouse. Participants were told to fixate a cross prior to each trial, which was displayed in the middle of the screen for 500 ms, and were encouraged to react as fast as possible after detecting the emotional target face.

### Results

To determine whether young target faces were recognized more quickly and more accurately as a function of target sex and emotion expression than old faces, mean accuracies and log transformed response times were computed for each combination of target age, target sex and target emotion. For response time, only correct responses above 200 ms were log transformed and included in the analyses. However, raw values are given in the text and in the figures to facilitate interpretation of the data. Response time and accuracy were then analyzed in separate three-way analyses of variance (ANOVA), with target age (young, old), target sex (male, female), and target emotion (happy, angry, sad) as within-subjects factors. Where Mauchly’s test indicated the violation of sphericity, Greenhouse Geisser corrections were applied and degrees of freedom were rounded to the next integer.

It should also be noted, that in an initial analysis, response time outliers, defined as deviating more than 3 SD from an individuals’ mean (1.7% of trials), were excluded. However, both analyses revealed the same effects and thus, the statistic values are shown for response times including those outliers.

#### Accuracy

**FIGURE 3:**
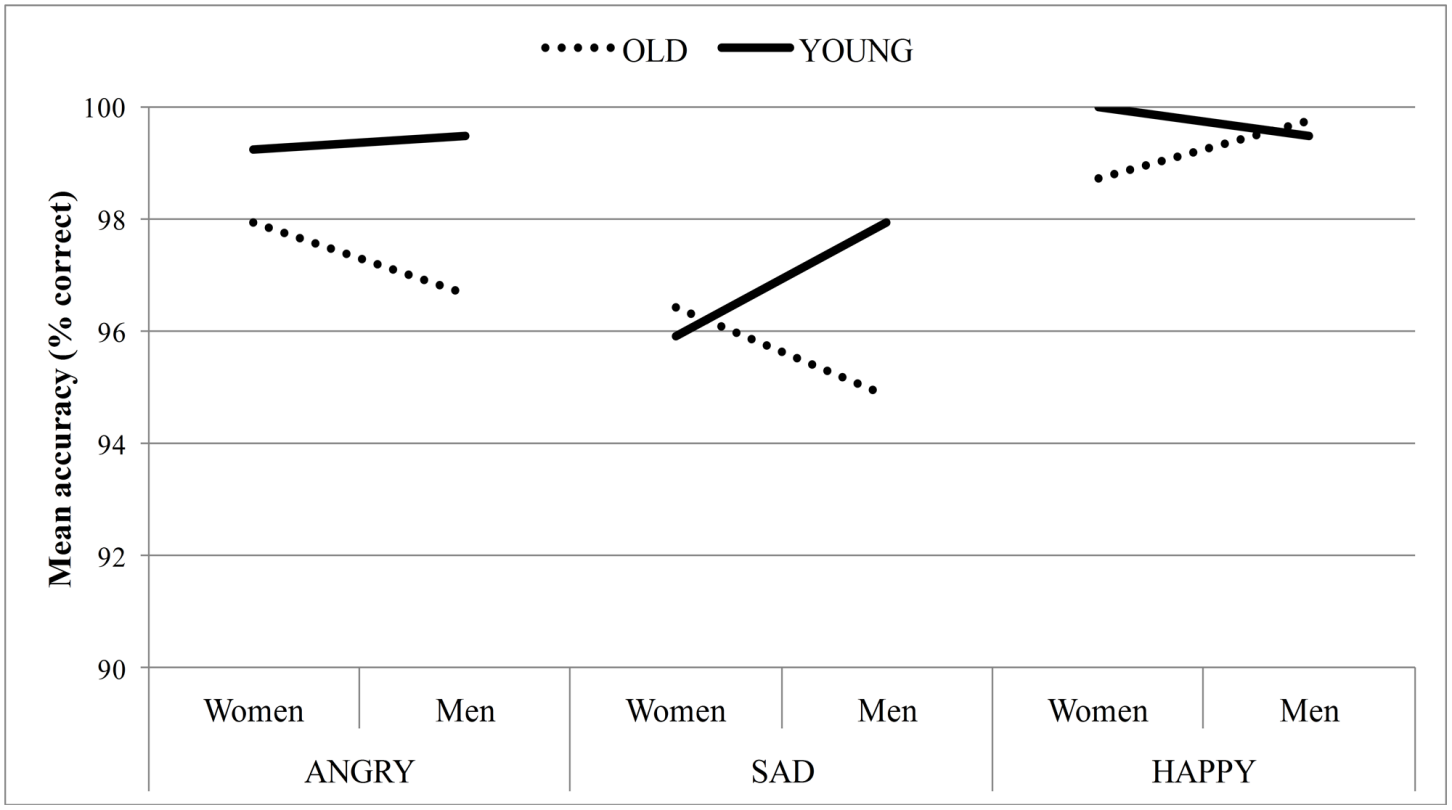
**Mean accuracy of target detection as a function of target age, sex, and emotion**.

Overall, participants were very good at locating the emotional target face (*M* = 98%, *SD* = 2.40). A main effect of emotion, *F*(2,84) = 9.16, *p* < 0.001, $\eta_{p}^{2}$ = 0.18, emerged, such that decisions were made more accurately for angry (*M* = 98%, *SE* = 0.57) and happy targets (*M* = 99%, *SE* = 0.19) relative to sad targets (*M* = 96%, *SE* = 0.78). As predicted, a significant main effect of target age, *F*(1,42) = 12.37, *p* = 0.001, $\eta_{p}^{2}$ = 0.23, emerged. This main effect was qualified by a significant target age × sex × emotion interaction, *F*(2,67) = 3.67, *p* = 0.04, $\eta_{p}^{2}$ = 0.08 (see **Figure 3**). No other effect was significant. Simple effects analyses were conducted in the form of separate ANOVAs on angry, sad and happy face blocks. When the target face expressed anger, decisions were made more accurately on young (*M* = 99.36%, *SE* = 0.42) compared to old faces (*M* = 97.31%, *SE* = 0.81), *F*(1,42) = 12.47, *p* = 0.001, $\eta_{p}^{2}$ = 0.23. Neither the main effect of sex nor the interaction between target age and sex reached significance for angry targets. For sad and happy targets, no main effect of target age or sex emerged, but a marginal significant interaction between target age and sex was found [sadness: *F*(1,42) = 3.45, *p* = 0.07, $\eta_{p}^{2}$ = 0.08; happiness: *F*(1,42) = 3.84, *p* = 0.06, $\eta_{p}^{2}$ = 0.08]. Post hoc paired *t*-tests revealed that sadness was recognized more accurately on young men (*M* = 98%, *SD* = 4.95) compared to old men (*M* = 95%, *SD* = 9.40), *t*(42) = -2.492, *p* = 0.02. In line with the age trend, happiness was also recognized more accurately on young women (*M* = 100%, *SD* = 0.00) compared to old women (*M* = 99%, *SD* = 3.57), *t*(42) = -2.351, *p* = 0.02.

Thus, compared to old target faces, young target faces expressing anger were detected more accurately. Findings regarding sad and happy facial expressions were less consistent, but when there were age differences, young target faces were detected more accurately than old target faces. Therefore the hypothesis that emotional expressions are perceived more accurately in the young relative to the old face appears to receive strong support in terms of the accuracy criterion.

#### Response Time

**FIGURE 4:**
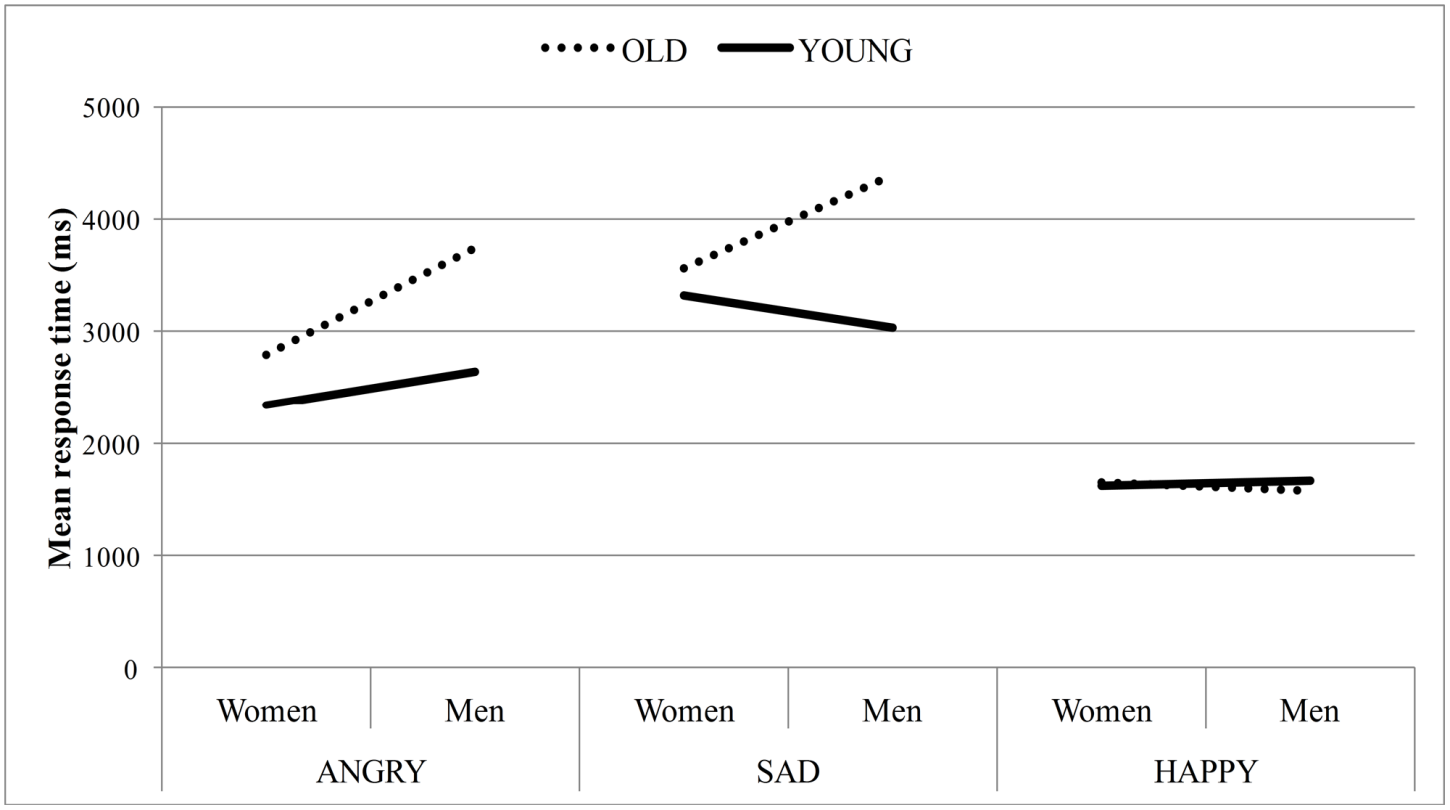
**Mean response time for correct responses as a function of target age, sex, and emotion**.

The analysis of the log transformed response time revealed a main effect of emotion, such that in a group of neutral faces happy targets (*M* = 1,623, *SE* = 67.13) were detected earlier than angry faces (*M* = 2,883, *SE* = 128.00), which were recognized faster than sad targets (*M* = 3,583, *SE* = 220.30), *F*(2,84) = 190.36, *p* < 0.001, $\eta_{p}^{2}$ = 0.82. The main effects of age and sex, as well as all two-way interactions were qualified by the three-way interaction between target age, sex and emotion, *F*(2,84) = 19.49, *p* < 0.001, $\eta_{p}^{2}$ = 0.32. Specifically, the difference in response time for young and old targets varied as a function of target sex and emotion (see **Figure 4**) [age: *F*(1,42) = 96.73, *p* < 0.001, $\eta_{p}^{2}$ = 0.70; sex: *F*(1,42) = 38.51, *p* < 0.001, $\eta_{p}^{2}$ = 0.48; age × sex: *F*(1,42) = 24.90, *p* < 0.001, $\eta_{p}^{2}$ = 0.37; age × emotion: *F*(2,84) = 30.81, *p* < 0.001, $\eta_{p}^{2}$ = 0.42; sex × emotion: *F*(2,84) = 27.83, *p* < 0.001, $\eta_{p}^{2}$ = 0.40).

Simple effects analyses were conducted. A 2(age) × 2 (sex) ANOVA on angry faces revealed main effects for target age and sex, modified by the target age × sex interaction, *F*(1,42) = 20.37, *p* < 0.001, $\eta_{p}^{2}$ = 0.33 [age: *F*(1,42) = 66.03, *p* < 0.001, $\eta_{p}^{2}$ = 0.61; sex: *F*(1,42) = 76.95, *p* < 0.001, $\eta_{p}^{2}$ = 0.65]. Follow up paired *t*-tests revealed, that young angry faces (men: *M* = 2,647, *SD* = 716; women: *M* = 2,344, *SD* = 675) were detected faster than old angry faces (men: *M* = 3,748, *SD* = 1,340; women: *M* = 2,793, *SD* = 954) and this effect was larger for male faces [male target: *t*(42) = 8.933, *p* < 0.001; female targets: *t*(42) = 4.230, *p <* 0.001]. Also, women’s angry faces were detected faster than men’s angry faces and this effect was larger for old faces [old face: *t*(42) = -9.207, *p* < 0.001; young face: *t*(42) = -3.457, *p* = 0.001].

The two-way ANOVA on sad faces revealed a main effect for target age, *F*(1,42) = 52.87, *p* < 0.001, $\eta_{p}^{2}$ = 0.56, such that young sad faces were detected earlier than old sad faces. Although the main effect of target sex was not significant, the interaction between target age × sex indicated that the difference in response time between female and male sad targets varied as a function of age, *F*(1,42) = 26.90, *p* < 0.001, $\eta_{p}^{2}$ = 0.39. Whereas correct decisions for sad old women (*M* = 3,569, *SD* = 1,411) were made faster than for sad old men (*M* = 4,395, *SD* = 1,961), *t*(42) = -4.972, *p* < 0.001, correct decisions for sad young women (*M* = 3,324, *SD* = 1,674) were made less rapidly than for sad young men (*M* = 3,043, *SD* = 1,158), *t*(42) = -2.949, *p* = 0.005.

The two-way ANOVA on happy faces, revealed no significant main effects of target age or target sex. However, there was a significant interaction between target age and sex, *F*(1,42) = 7.78, *p* = 0.008, $\eta_{p}^{2}$ = 0.16. Whereas there was no difference in mean response time for old happy women (*M* = 1,650, *SD* = 425) compared to young happy women (*M* = 1,613, *SD* = 465), *t*(42) = 0.829, *p* = 0.412, happy old men (*M* = 1,571, *SD* = 488) were detected even faster than happy young men (*M* = 1,659, *SD* = 499), *t*(42) = -2.854, *p* = 0.007. Furthermore, old happy men were detected earlier than old happy women, *t*(42) = 2.332, *p* = 0.025, with no such difference for young happy targets, *t*(42) = -1.424, *p* = 0.162.

Thus, compared to old target faces, young target faces expressing anger or sadness were detected faster. However, this was not the case for happy facial expressions. Therefore, the hypothesis that emotional expressions are perceived faster in the young relative to the old face appears to receive strong support in terms of response time only for negative emotions.

### Discussion

The primary purpose of Experiment 2 was to assess whether face age impairs the recognition of emotional targets among a neutral set of faces of same sex peers in a visual search task. Our results suggest that emotional young faces are more quickly detected than emotional old faces, as reflected by overall faster response times for an emotional young face among neutral distractors relative to an emotional old face among neutral old distractors. In addition, participants were also better at identifying angry and sad targets in young neutral groups relative to old neutral groupings. However, the age of the face did not impair the identification of a happy face consistently, but research has shown that happiness is the most easily decoded expression.

Despite high accuracies across all three facial expressions, the difference in response time varied considerably. Ever since Hansen and Hansen (1988) reported that angry facial expressions are found more efficiently than happy facial expression, this design has been a focus of contention. The original study suffered from methodological confounds, but the implications of other methodological choices in this design have been contentious as well (Purcell and Stewart, 2010; Becker et al., 2011; Craig et al., 2014). Criticism has focused mainly on the degree to which the results of these studies represent responses to the displayed emotional expression *per se*, or rather are driven by low-level visual features that are unrelated to facial affect (Pinkham et al., 2010; Purcell and Stewart, 2010; Craig et al., 2014). In all, there is mixed evidence whether happy or angry facial expressions are processed more efficiently (see Shasteen et al., 2014; Lipp et al., 2015). Nonetheless, we decided to use this paradigm in the present research. To that effect, it should be noted that we were not interested in assessing whether affectively positive or negative stimuli are processed preferentially. Rather, we were interested in differences in emotion recognition as a function of face age. As happiness can be characterized by a single salient feature anything but a fast and accurate emotion recognition would be surprising (Adolphs, 2002). Thus, as noted above, it is likely that the low task difficulty for happy faces reduced the effect of face age. Specifically, the open smile is a salient feature that is easily discernable among the distractor faces without a detailed analysis of other facial features. Consequently, there is no search advantage for young relative to old faces in the emotion recognition of happy faces. And this is what we found. However, when multiple facial features have to be extracted, as in sad and angry facial expressions, the level of task difficulty, and consequently the influence of distractor faces increases. We argue that neutral young faces can easily be grouped together as distractors, whereas neutral old faces need additional processing resources as facial morphological features have to be discriminated from facial expressive features.

Apart from low-level features as a source of pop-out effects, the literature on visual search tasks also suggests that familiarity influences visual search performance. A slowed search occurs especially when attention is captured by unfamiliar distractors (Wang et al., 1994; Malinowski and Hübner, 2001; Shen and Reingold, 2001). Thus, the level of contact with the elderly could account for age-related differences in visual search performance. Also, a differential motivation to respond to young relative to old individuals might explain differences in emotion recognition between young and old faces (Malatesta et al., 1987b; Lamy et al., 2008). However, as our sample included both younger and older individuals neither explanation seems satisfactory.

The identification of the target emotion is also influenced by expectations. This is why standard visual search designs usually just demand a decision as to whether all faces are the same or one is different. One might therefore argue that informing participants about the target stimulus’ type of emotional expression before the search, serves as artificial priming that does not occur in real-world settings (Pinkham et al., 2010). However, emotion perception in an interpersonal situation does not occur in a social vacuum. Instead, there might be more information on the emotional state of the interactional partner, as, e.g., voice tone (Kappas et al., 1991), knowledge about private matters of the other or even earlier emotional interactions with that person (Hess and Hareli, 2014). Those cues regarding the likely emotional state of others are typically available to the perceiver before emotional features in the face are noticed. Yet, although the repetition of the same emotion on successive trials speeds search (Lamy et al., 2008), the variation of target age within an emotion block makes emotional priming also an unlikely explanation for differences in emotional target discrimination in young and old face groups.

As slowness is a quality stereotypically associated with elderly people and the activation of elderly stereotypes can result in behavior in line with this stereotype such as slower walking speed (Bargh et al., 1996), the presentation of older target faces might activate such stereotypes and therefore prime slower responses in general. However, in our design the target age switched randomly from trial to trial to avoid accumulated priming effects. Further, no such slowing influence of target age was found for happy facial expressions, which also supports our interpretation focused on task difficulty.

One could question the practical significance of these results, especially the size of the age-related differences in the accuracy rates. Generally, a mean accuracy rate of 98% seems very good. On the other hand, the task difficulty was also comparably low. Without time restriction, participants simply had to pick an emotional face among neutral faces. This was further facilitated in that all emotional photographs depicted individuals with a high level of emotion intensity. It should be noted that, participants made more mistakes in the case of older relative to younger targets and were more than a second slower for the old targets in correct trials. Imaging a situation that is closer to real life, the emotional display would probably appear less clear due to a lower emotion intensity and may occur in a more ambiguous context.

In sum, whereas previous studies on impaired emotion identification in the old face used individually presented still photos or videos with single actors, we investigated emotion recognition in a simulated social context. In each display, only one identity varied from the same sex peers by emotional content, whereas all faces differed from one another by virtue of face identity. Thus, distractor faces offered great heterogeneity and increased the difficulty of finding the discrepant stimulus. In such a design, we found slowed and less accurate visual search to detect emotional old faces relative to young faces for negative emotions. We conclude that the visual properties in the aged face, specifically the presence of wrinkles and changes in facial appearance that are age-related, are the most plausible explanation for this pattern of results.

## General Discussion

The current article addressed in three experiments how the age of a face impairs the perception of emotional expressions. Experiment 1A,B revealed that misattributions of emotions to neutral faces are more likely for old stimuli than for young individuals. Specifically, participants rated neutral old faces not only as angrier and, for male faces, as sadder than neutral young faces, but also as happier. Further, Experiment 2 tested whether this perceived “emotionality” in old faces hindered emotion identification in a visual search task. In fact, we found a search disadvantage for emotional old faces among neutral same-sex peers, likely due to the attentional capture by ambiguous facial displays, especially for negative emotions.

Setting both experiments in context, more anger was attributed to neutral faces of old individuals in the human ratings and likewise participants spent more time and made more errors in trials when they had to find an angry old face among a set of same-aged neutral faces. Simultaneously, more sadness was attributed to neutral faces of old men and sad facial expressions were found less accurately relative to young men in groups of the same sex and age. With regards to response time, participants were generally slower for old compared to young faces.

Our results strike us as especially interesting in light of the large age range of the perceivers. Specifically, we regard our heterogeneous sample as advantageous, particularly for Experiment 2 with an age up to 65 years. This allows us to conclude that the differences in emotion perception in young and old faces described in the current article did not result from an own-age advantage by young participants, as could be argued. Moreover, less contact with the elderly that may lead to deficits in emotion decoding also affects our findings less than would be the case with a student sample. For a further discussion on how the age congruence between an observer and face might influence facial expression decoding, see Fölster et al. (2014).

A promising issue for future research regards the configural processing of faces. An increased motivation to process ingroup compared to outgroup faces explains, in part, the ingroup advantage in expression identification (Thibault et al., 2006; Young and Hugenberg, 2010). Young and Hugenberg (2010) further argue that configural processing of ingroup faces drives this advantage, which disappears after face inversion. Given that the recognition of happy faces is unaffected by face inversion (McKelvie, 1995; but see also Leppänen and Hietanen, 2007), it seems reasonable to pose the question, whether the distracting wrinkles in the elderly face lead to a more feature-by-feature processing of the old face. If this were the case we would indeed expect happy faces to be spared. Hence, it would be interesting to investigate whether impaired recognition in still photos of old relative to young faces not only occurs in upright, but also inverted faces, where configural processing of young face would also be disturbed.

As noted earlier, the fact that emotional signals by older individuals are perceived with less clarity has potential implications for our social lives. Generally, it seems critically relevant to the quality of social interactions to reduce misinterpretations of emotional expressions. Emotions occur between people (Fischer and van Kleef, 2010) and influence social relationships. The accurate perception of emotion displays and emotional states helps to coordinate and facilitate interpersonal interaction and communication (Keltner and Haidt, 2001; Niedenthal and Brauer, 2012) and provides the necessary “affective glue” between individuals (Feldman et al., 1991). Given the literature on the relationship between loneliness and depressive symptoms in the elderly (see Hawkley and Cacioppo, 2010, for review), the impaired recognition of facial emotions expressed by elderly people is especially problematic, because the relationship between loneliness and depression in the elderly is mediated by social support (Liu et al., 2014). Following this line of argument, a lack of signal clarity in the elderly face can result in emotional misunderstandings and hence dysfunctional behavior from the environment, such that the intended social support will not be perceived as supportive.

In sum, the present research provides further evidence for the notion that the emotional expressions of the elderly may easily be misunderstood. Specifically, because neutral expressions already seem “emotional” two problems may occur. On one hand, elderly people may be perceived as expressing (negative) emotions when in fact they are not, and conversely, their (negative) emotions may not be perceived as such when shown. This may be the case especially when surrounded by others. This has implications for everyday life, especially in contexts where many elderly people are present, such as in nursing homes.

## Conflict of Interest Statement

The authors declare that the research was conducted in the absence of any commercial or financial relationships that could be construed as a potential conflict of interest.
