# Supplementary material for: Through a glass darkly: facial wrinkles affect our processing of emotion in the elderly
Source: Front Psychol. 2015 Oct 1;6:1476. doi: 10.3389/fpsyg.2015.01476 (PMC4589643; doi:10.3389/fpsyg.2015.01476)
Supplement: Supplementary file 1 [file Table_1.PDF]

Table 1: Correlation Table

Summary of Pearsons correlation coefficients *r* comparing human ratings and the automated assessment by CERT for each emotion. Different levels of significance are marked with asterisks. For ease of comparison, significant positive correlation (blue values) and significant negative correlations (red values) are highlighted.

***The correlation is significant at the .01 level (2-tailed).
**The correlation is significant at the .05 level (2-tailed).
*The correlation is significant at the .10 level (2-tailed).

Correlations (Pearson)		Human Rating							CERT Probability Estimates								Mean	SD	N
		Anger	Contempt	Disgust	Fear	Happiness	Sadness	Surprise	Anger	Contempt	Disgust	Fear	Happiness	Sadness	Surprise	Neutral Display			
Human Rating	Anger																1.06	.577	36
	Contempt	.850***															1.13	.423	36
	Disgust	.586***	.753***														0.56	.334	36
	Fear	-.111	-.240	.010													0.71	.347	36
	Happiness	-.417**	-.317*	-.343**	-.478***												0.48	.705	36
	Sadness	-.064	-.071	.099	.740***	-.455***											1.38	.611	36
	Surprise	-.121	-.269	.003	.376**	-.029	-.035										0.58	.345	36
CERT Probability Estimates	Anger	.315*	.066	-.147	-.213	.051	-.152	-.277									12,96%	.184	36
	Contempt	-.217	-.040	-.035	-.423**	.318*	-.334**	-.169	-.237								14,98%	.134	36
	Disgust	.331**	.169	-.024	-.060	-.012	-.057	-.036	.260	-.349**							5,25%	.112	36
	Fear	-.126	-.080	.051	.364**	.033	.089	.078	-.206	-.178	-.113						3,19%	.067	36
	Happiness	-.171	-.094	-.053	-.319*	.831***	-.345**	.145	-.014	.103	.092	.144					2,41%	.054	36
	Sadness	.156	.208	.270	.286*	-.066	.309*	-.045	-.092	-.444***	-.022	.105	.015				18,46%	.217	36
	Surprise	-.215	-.240	-.129	.401**	-.124	.288*	.015	-.262	-.052	-.184	.595***	-.112	-.041			0,96%	.022	36
	Neutral Display	-.297*	-.212	-.084	.094	-.309*	.071	.284*	-.539***	.201	-.391**	-.138	-.329*	-.555***	.111		41,79%	.261	36
